# Briefing in Application of Machine Learning Methods in Ion Channel Prediction

**DOI:** 10.1155/2015/945927

**Published:** 2015-04-16

**Authors:** Hao Lin, Wei Chen

**Affiliations:** ^1^Key Laboratory for Neuro-Information of Ministry of Education, Center of Bioinformatics, School of Life Science and Technology, , Chengdu 610054, China; ^2^Department of Physics, School of Sciences and Center for Genomics and Computational Biology, Hebei United University, Tangshan 063000, China

## Abstract

In cells, ion channels are one of the most important classes of membrane proteins which allow inorganic ions to move across the membrane. A wide range of biological processes are involved and regulated by the opening and closing of ion channels. Ion channels can be classified into numerous classes and different types of ion channels exhibit different functions. Thus, the correct identification of ion channels and their types using computational methods will provide in-depth insights into their function in various biological processes. In this review, we will briefly introduce and discuss the recent progress in ion channel prediction using machine learning methods.

## 1. Background

Ion channels are a diverse group of proteins that extend across the lipid membrane of cells and form channel pores [1]. They allow ions to move into and out of the cell to establish and control the voltage gradient across the cell membrane in response to stimuli, such as ligand, voltage, and pressure changes. Many biological processes including muscle contraction, neuronal excitability, epithelial transport of nutrients and ions, hormone secretion, T-cell activation, and pancreatic beta-cell insulin release are all controlled and regulated by ion channels [2].

It has been reported that the normal function of ion channels can be disrupted by chemicals and genetics, which would result in negative impact on the organism [2]. For example, channelopathies are caused by mutations in ion channel-encoding genes [3]. Moreover, various neurotoxins bind to ion channels to modulate the nervous systems of animals. Since ion channels have such important biological function in various biological processes, scientists have developed drugs to target them for disease therapy. Ion channels have been demonstrated as valuable targets for the treatment of epilepsy, chronic pain, and other diseases [4].

Over 300 types of ion channels have been found in living cells [5]. Most channels are ion-selective and ion-specific. For example, most of potassium channels have a permeability ratio for potassium over sodium of 1000 : 1 [6]. Based on their biological properties, ion channels can be clustered into numerous types. The ion channels activated by the binding of ligand molecules (such as a neurotransmitter) are called ligand-gated ion channels (LGIC) that can be further classified into three superfamilies, namely, Cys-loop receptors, ionotropic glutamate receptors, and ATP-gated channels. Voltage-gated ion channels (VGIC) are another kind of ion channels which open to allow ions to pass through the membrane in response to the changes in electrical potential difference. According to ion type permeability, the VGICs can be further classified into potassium (K), sodium (Na), calcium (Ca), and anion VGICs. Moreover, some ion channels can also be opened and closed by mechanical forces, temperature, and pressure. However, the number of these ion channels is too few to have statistical significance. Thus, this review focuses on the prediction of ligand-gated and voltage-gated ion channels.

Different ion channel types perform different biological functions and regulate different biological possesses. To identify the types of ion channels, traditional biochemical experimental methods are time-consuming and costly, whereas computational methods are cost-effective. Therefore, in this paper, we review the development of machine learning methods in the prediction of ion channel and their types. To predict ion channels using machine learning method, the following issues should be considered. (i) How to construct a valid and objective benchmark data set to train and test the predictor? (ii) How to formulate the ion channel sequences using an effective mathematical descriptor which can truly reflect the properties of samples? (iii) How to develop or use a machine learning algorithm to perform the prediction? (iv) What kind of cross-validation tests should be used to evaluate the anticipated accuracy of the predictor? We will discuss each issue in turn.

## 2. Published Databases

The first essential requirement in developing computational methods for the prediction of ion channels is to obtain a benchmark database. At present, many public databases are available online. Some of these original databases, such as protein data bank (PDB) [7] and universal protein resource (UniProt) [8], have deposited many ion channel data. Based on these databases and related publications, some special databases such as IUPHAR (International Union of Basic and Clinical Pharmacology) database [9], ligand-gated ion channel database [10], and VKCDB [11] have been built. The web addresses of these databases are listed in Table 1.

However, the databases listed above are not suitable for ion channel prediction using machine learning methods, because the data deposited are redundant and are of low quality. A reliable and objective benchmark dataset should be constructed by the following strategies: (i) if the protein sequence of an ion channel contains ambiguous residues (such as “B,” “X,” and “Z”), the ion channel must be excluded; (ii) if sequences are fragments of other proteins, the sequences must be excluded; (iii) if an ion channel is inferred from homology or prediction, the ion channel must also be excluded; and (iv) the highly similar sequences must be excluded for objectivity, because the high similarity data will lead to overestimating the performance of the proposed predictors.

In order to exclude highly similar sequences from these datasets, BLASTClust, CD-HIT [12], and PISCES [13] have been developed and could be freely obtained at the addresses listed in Table 2. BLASTClust is a program that can be used to cluster either protein or nucleotide sequences. However, since it requires all against all comparisons of sequences for optimal results [14], the efficiency of this program is relatively low. Owing to the clustering efficiency and capability to handle extremely large databases, CD-HIT has been widely employed to remove redundant sequences. However, CD-HIT cannot deal with sequences with sequence identity below 40%. To overcome this shortcoming, PISCES was proposed in 2003, which can exclude proteins with the sequence identity of 25% [13].

According to the above mentioned public databases and sequence culling programs, four benchmark datasets of ion channels have been proposed in previous studies [15–19].

The first benchmark dataset S1 [19] contains 1574 nonion channels and 473 ion channels, of which 164 are potassium, 27 sodium, 27 calcium, and 18 chloride VGICs. The sequence identity between any two sequences in S1 is less than 90%. These data are derived from the Swiss-Prot database.

The second nonredundant benchmark dataset S2 [17] contains 37 Kv1, 16 Kv2, 18 Kv3, 15 Kv4, and 14 Kv7 subfamilies of voltage-gated K+ channels. These data are derived from the VKCDB database.

The third benchmark dataset S3 [16] contains 300 nonion channel membrane proteins and 298 ion channel proteins. The ion channel dataset contains 148 VGICs (81 potassium, 29 calcium, 12 sodium, and 26 anion VGICs) and 150 LGICs. The sequence identity of this dataset is less than 40%. These data are derived from the Uniprot and LGIC databases.

The fourth benchmark dataset S4 [15] contains 217 voltage-gated K+ channels, composed of 82 Kv1, 16 Kv2, 37 Kv3, 32 Kv4, 10 Kv6, and 40 Kv7 families, respectively. The sequence identity of this dataset is less than 60%. These data are derived from the VKCDB database.

## 3. Methods

### 3.1. Protein Description

Use of informative parameters to represent the ion channel samples is the second essential requirement for bioinformatics prediction. Here, three kinds of features, amino acid compositions, dipeptide compositions, and tripeptide compositions, were used to represent ion channels and expressed as follows:

(1) $$\begin{array}{c} f_{20}\left(i\right)=\frac{x_{20}\left(i\right)}{\sum_{i}x_{20}\left(i\right)}, \end{array}$$

(2) $$\begin{array}{c} f_{400}\left(j\right)=\frac{y_{400}\left(j\right)}{\sum_{j}y_{400}\left(j\right)}, \end{array}$$

(3) $$\begin{array}{c} f_{8000}\left(k\right)=\frac{z_{8000}\left(k\right)}{\sum_{k}z_{8000}\left(k\right)}, \end{array}$$

where *x*_20_(*i*), *y*_400_(*j*), and *z*_8000_(*k*) are the occurrence number of residues *i*, the number of occurrences of dipeptide *j*, and the number of occurrences of tripeptide *k* in the protein sequence of an ion channel, respectively. 20, 400, and 8000 are the number of the standard amino acids, the number of combination of dipeptides, and the number of combination of tripeptides, respectively.

### 3.2. Feature Selection

Theoretically, high dimension features will lead to three serious issues, that is, overfitting, information redundancy, or noise and dimension disaster [20]. These issues would result in low generalization ability of the predictor, poor prediction accuracy, and time-consuming computations. Thus, it is necessary to use feature selection techniques to optimize feature set for economizing the time for computation and building robust prediction models. In the following section, we will discuss how to use three feature selection techniques, that is, analysis of variance, correlation-based feature selection, and binomial distribution, to select optimal features.

#### 3.2.1. Analysis of Variance (ANOVA)

To evaluate the contribution of features to the classification, the *F* value (*F*(*λ*)) of the *λ*th feature can be defined as

(4) $$\begin{array}{c} F\left(\lambda \right)=\frac{s_{B}^{2}\left(\lambda \right)}{s_{W}^{2}\left(\lambda \right)}, \end{array}$$

where *s*_*B*_^2^(*λ*) is called means square between (MSB) and denotes the sample variance between classes and *s*_*W*_^2^(*λ*) is called mean square within (MSW) and denotes sample variance within classes. They can be calculated by [16]

(5) sB2λ =∑i=1Kni∑j=1nifijλ/ni−∑i=1K∑j=1nifijλ/∑i=1Kni2dfB,

(6) $$\begin{array}{c} s_{W}^{2}\left(\lambda \right)=\frac{{\sum_{i=1}^{K}\sum_{j=1}^{n_{i}}\left(f_{ij}\left(\lambda \right)-\left(\sum_{j=1}^{n_{i}}f_{ij}\left(\lambda \right)\right)/n_{i}\right)}^{2}}{df_{W}}, \end{array}$$

where *K* and *N* represent the number of classes and total number of samples, respectively. *f*_*ij*_(*λ*) represents the frequency of the *λ*th feature of the *j*th sample in the *i*th class. *n*_*i*_ is the number of samples in the *i*th class. *df*_*B*_ = *K* − 1 is the degrees of freedom for MSB and *df*_*W*_ = *N* − *K* the degrees of freedom for MSW.

Based on the theory of statistics, the *F*(*λ*) in (4) obeys *F* sampling distribution with degrees of freedom *df*_*B*_ and *df*_*W*_. The *F*(*λ*) measures the contribution of the *λ*th feature related to the class variables. In the absence of differences between groups, the *F*(*λ*) will be close to 1. In other words, the feature with a larger *F*(*λ*) indicates that it is a more highly relevant one for the target to be predicted. Thus, features can be initially ranked according to *F* value.

#### 3.2.2. Correlation-Based Feature Selection (CFS)

The heart of the correlation-based feature selection algorithm is to evaluate the merit of a feature subset and exclude the redundant features which are highly correlated with one or more of the other features. The merit of a feature subset *S* containing *k* features is defined by the following equation [15]:

(7) $$\begin{array}{c} \text{Merit}\left(S,k\right)=\frac{k\bar{r_{cf}}}{\sqrt{k+k\left(k-1\right)\bar{r_{ff}}}}, \end{array}$$

where $\bar{r_{cf}}$ is the average feature-class correlation expressed as in the following equation and $\bar{r_{ff}}$ the average feature-feature intercorrelation, which can be defined as

(8) $$\begin{array}{c} \bar{r_{cf}}=\frac{1}{k}\underset{f_{z}\in \theta }{\sum }r_{cf_{z}},\\ \bar{r_{ff}}=\frac{2}{k\left(k-1\right)}\underset{f_{i},f_{j}\in \theta }{\sum }r_{f_{i}f_{j}}, \end{array}$$

where *c* is the class. The numerator in (8) indicates predictive ability of subset *θ* and the denominator stands for redundancy among the features. In fact, (7) is the Pearson's correlation where all variables have been normalized. The numerator gives an indication of how predictive a group of features are, whereas the denominator describes how much redundancy there is among them.

#### 3.2.3. Binomial Distribution (BD)

For a stochastic event, two possible cases, namely, occurrence and nonoccurrence, will happen when one observes the *i*th feature occurring in the *k*th type set [18]. Each outcome has a fixed probability when benchmark dataset has been fixed. This probability is called prior probability *p*_*k*_.

The total occurrence number of the *i*th feature in benchmark dataset is expressed as *N*_*i*_. That is to say, under the condition of the prior probability *p*_*k*_, one performs trial or observation with *N*_*i*_ times. The posterior probability *P*_*ik*_ of the *i*th feature occurring *n*_*ik*_ or more times in the *k*th type set can be calculated as follows

(9) $$\begin{array}{c} P_{ik}=1-CL_{ik}=\overset{N_{i}}{\underset{m=n_{ik}}{\sum }}\frac{N_{i}}{m!\left(N_{i}-m\right)!}p_{k}^{m}{\left(1-p_{k}\right)}^{N_{i}-m}, \end{array}$$

where *CL*_*ik*_ is the confidence level (*CL*) of the *i*th feature in the *k*th dataset. Based on small probability event principle, if *P*_*ik*_ is a small value, it means the feature *i* appearing in dataset *k* is not random. The feature with a small *P*_*ik*_ indicates that it is a more highly relevant one for the target to be predicted. Thus, features can be initially ranked according to *P*_*ik*_ value.

The incremental feature selection (IFS) can be used to determine the optimal number of features. The IFS procedure includes the following steps: starting with one feature with the first score in the feature set, adding the second feature with the second score, adding the third feature with the third score, and repeating this process until all candidate features are added. Finally, the proposed machine learning methods are used to investigate the performance of each feature subset. The feature subset which can yield the maximum accuracy is the optimal feature subset.

### 3.3. Support Vector Machine (SVM)

The third essential key for bioinformatics is to select an efficient and accurate machine learning method to make a predictive decision. SVM is a kind of machine learning method which has been successfully used in wide fields of ion channel prediction. Many researchers have developed free and convenient software packages for the implementation of SVM, such as LibSVM [21] and SVM_Light [22].

The basic idea of the SVM is described as follows. For a two-class classification problem, a series of training vectors $\vec{X_{i}}\in R^{d}\;\;(i=1,2,\ldots ,N)$ with corresponding labels *y*_*i*_ ∈ {+1, −1}  (*i* = 1,2,…, *N*) can be generated. Here, +1 and −1, respectively, indicate the two classes. SVM maps the input vectors $\vec{X_{i}}\in R^{d}$ into a high dimensional feature space in order to construct an optimal separating hyperplane with the largest distance between the two classes. The decision function implemented by SVM is written as

(10) $$\begin{array}{c} f\left(\vec{X}\right)=\mathrm{sgn}\left(\overset{N}{\underset{i=1}{\sum }}y_{i}\alpha_{i}\cdot K\left(\vec{X},\vec{X_{i}}\right)+b\right), \end{array}$$

where $K(\vec{X},\vec{X_{i}})$ is a kernel function which defines an inner product in a high dimensional feature space. There are three kinds of kernel functions for the nonlinear classification problems defined as follows.

 Polynomial function
  (11) $$\begin{array}{c} K\left(\vec{X_{i}},\vec{X_{j}}\right)={\left(\vec{X_{i}}\cdot \vec{X_{j}}+1\right)}^{d}. \end{array}$$
 Radial basis function (RBF)
  (12) $$\begin{array}{c} K\left(\vec{X_{i}},\vec{X_{j}}\right)=\exp\left(-\gamma {\left\|\vec{X_{i}}-\vec{X_{j}}\right\|}^{2}\right). \end{array}$$
 Sigmoid function
  (13) $$\begin{array}{c} K\left(\vec{X_{i}},\vec{X_{j}}\right)=\tanh\left[b\left(\vec{X_{i}}\cdot \vec{X_{j}}\right)+c\right]. \end{array}$$

The coefficients *α*_*i*_ can be solved by the following convex quadratic programming (QP) problem:

(14) Maximize ∑i=1Nαi−12∑i=1N∑j=1Nαiαj·yiyj·KXi→,Xj→subject  to 0≤αi≤C,

where ∑_*i*=1_^*N*^ *α*_*i*_ *y*_*i*_ = 0, *i* = 1,2,…, *N*. The regularization parameter *C* can control the trade-off between margin and misclassification error.

For multiclass problems, several strategies such as one-versus-rest (OVR), one-versus-one (OVO), and DAGSVM are applied to extend the traditional SVM. Because the RBF usually outperforms polynomial function and sigmoid function, the RBF is widely used in bioinformatics. The regularization parameter *C* and kernel parameter *γ* were tuned to optimize the classification performance using grid search with cross-validation.

### 3.4. Criteria for Performance Evaluation

In developing a useful statistical predictor, it is very important to objectively evaluate its performance or anticipated success rate. Here, a set of more intuitive and easier-to-understand metrics is introduced. Those are sensitivity (Sn), specificity (Sp), accuracy (Acc), and Matthew's correlation coefficient (MCC) defined as [23]

(15) $$\begin{array}{c} \text{Sn}=1-\frac{N_{-}^{+}}{N^{+}},\\ \text{Sp}=1-\frac{N_{+}^{-}}{N^{-}},\\ \text{Acc}=1-\frac{\left(N_{-}^{+}+N_{+}^{-}\right)}{\left(N^{+}+N^{-}\right)},\\ \text{MCC}=\frac{1-\left(N_{-}^{+}/N^{+}+N_{+}^{-}/N^{-}\right)}{\sqrt{\left(1+\left(N_{+}^{-}-N_{-}^{+}\right)/N^{+}\right)\left(1+\left(N_{-}^{+}-N_{+}^{-}\right)/N^{-}\right)}}, \end{array}$$

where *N* ^+^ is the total number of the positives while *N*_−_^+^ is the number of the positives incorrectly predicted as the negatives; *N* ^−^ is the total number of the negatives while *N*_+_^−^ is the number of the negatives incorrectly predicted as positives. These four metrics are generally used in statistical prediction for quantitatively measuring the performance of a predictor from four different angles.

Three cross-validation tests, that is, independent dataset test, subsampling (or *K*-fold cross-validation) test, and jackknife test, are often used to evaluate the anticipated success rate of a predictor [24]. The *K*-fold cross-validation is a kind of rigorous and objective method for evaluating the predictive performance of predictors. For *K*-fold cross-validation, the dataset is divided into *K* equal parts. Of these *K* parts, *K* − 1 parts are used for training and the *K*th part is used for testing. This process is repeated *K* times for all *K* parts and the success rate is the average of the *K* times tests. The jackknife test is deemed the least arbitrary one and hence has been widely used in the realm of bioinformatics. In the jackknife test, each sequence in the training dataset is in turn singled out as an independent test sample and all the rule-parameters (Sn, Sp, Acc, and MCC) are calculated without including the one being identified.

## 4. Published Results

Although many works have investigated the dynamics of ion channel, only few pattern recognition methods focused on the prediction of ion channels. The pioneering works for the prediction of ion channels were carried out independently by two groups in 2006.

Based on the benchmark dataset S1, a SVM-based method (SVM_light package) was proposed to discriminate ion channels from nonion channels [19]. In five-fold cross-validation, the Accs of 82.89% and 85.56% were achieved by using amino acid composition (1) and dipeptide composition (2), respectively. Authors also investigated the performance of position-specific scoring matrix (PSSM) generated from PSI-BLAST (Position-Specific Iterative Basic Local Alignment Search Tool) which can provide the distant relationships between proteins. Three iterations of PSI-BLAST were carried out at a cut-off *E*-value of 0.01. Then the accuracy of 84.22% was obtained by using the five-fold cross-validation. By combining dipeptide composition with position-specific scoring matrix, the five-fold cross-validated accuracy increased to 89.11%. Subsequently, these methods were used to predict potassium, sodium, calcium, and chloride VGICs. For further improving the accuracy, the Hidden Markov model (HMM) profiles of the four types of VGICs were constructed using the HMMER software package. Each protein sequence was aligned in a multiple sequence alignment using ClustalW. The *E*-value threshold (*E*-value) was set to 0.01. As a result, the five-fold cross-validated Acc reached 97.78% by using the hybrid method that combines dipeptide-based SVM and hidden Markov model methods. The Sns (MCC) of potassium, sodium, calcium, and chloride VGIC predictions are 99.38% (0.96), 96.00% (0.93), 96.00% (0.98), and 86.67% (0.92). Based on these approaches, a web server VGIchan (http://www.imtech.res.in/raghava/vgichan/) was developed for predicting and classifying voltage-gated ion channels. This is the first online server for ion channel prediction using a machine learning method.

Based on the benchmark dataset S2, Liu et al. [17] predicted the five subfamilies of potassium VGICs by using SVM combined with dipeptide composition (2). In the jackknife cross-validation, the average Acc of 98.0% was achieved with the average Sn of 89.9%, Sp of 100%, and MCC of 0.94.

Although these two studies have achieved good results, the high sequence similarity of the two datasets might result in overestimating the performance and reducing the generalization ability of the proposed predictive models.

Recently, based on the benchmark dataset S3 and by using dipeptide composition (2) as parameters, Lin and Ding [16] successfully predicted ion channels and their types using Libsvm package. In jackknife cross-validation, the Accs of 85.0%, 89.9%, and 82.4% are obtained for the classification of ion channels and nonion channels, VGICs and LGICs, and the subclasses of VGICs, respectively. For further improving predictive performance of SVM model, the ANOVA (3)–(5) was firstly proposed to select the optimal dipeptide compositions (2). Then, the Accs increase from 85.0%, 89.9%, and 82.4% to 86.6%, 92.6%, and 87.8%, respectively, when using the 140, 159, and 232 optimal dipeptides according to the *F* values, respectively. These results demonstrate that the ANOVA is a powerful and efficient feature selection technique which can improve the predictive accuracy by excluding noise and redundant parameters. Based on this proposed method, an effective tool for predicting ion channels and their types, called IonchanPred, was constructed and can be freely downloaded from http://cobi.uestc.edu.cn/people/hlin/tools/IonchanPred/. By using the IonchanPred, the KCMA1 can be correctly identified, which is a potassium channel activated by either membrane depolarization or increase in cytosolic Ca2+ and plays a key role in controlling excitability in a number of systems. For comparison, this feature selection technique was also used to investigate the performance of SVM on the benchmark dataset S1. In five-fold cross-validation, the Acc and average accuracy are 97.97% and 95.55%, respectively. Comparison demonstrates again that the ANOVA is a powerful technique for feature selection.

Based on the benchmark dataset S4, Chen and Lin presented a SVM-based method (LibSVM package) to predict six subfamilies of potassium VGICs using amino acid composition and dipeptide composition [15]. The Acc of 87.39% was achieved in jackknife cross-validation. Furthermore, the CFS was proposed to find the best feature set. As a result, the maximum Acc of 93.09% was obtained in jackknife cross-validation when 118 features were used. For the convenience of the vast majority of experimental scientists, a predictive tool, called VKCPred, was constructed and can be freely downloaded from http://cobi.uestc.edu.cn/people/hlin/tools/VKCPred/. For further improving the accuracy, Liu et al. [18] proposed BD-based feature selection technique to pick out optimal tripeptides. The LibSVM was used to execute the SVM algorithm. The overall accuracy improved to 96.77% in jackknife cross-validation when 648 tripeptides were selected as optimal features. A user-friendly web-server called iVKC-OTC was established and can be freely accessible at http://lin.uestc.edu.cn/server/iVKC-OTC.

The four tools, VGIchan, IonchanPred, VKCPred, and iVKC-OTC, are listed in Table 3 for use by experimental researches.

## 5. Prospect

Ion channels are important drug targets. Using computational methods can provide valuable information for narrowing the scope of drug targets discovery. However, few methods have been applied in this realm and the accuracy is still far from that required for successful application.

Many machine learning methods such as neural network (NN) [25], K nearest neighbor (KNN) [26], extreme learning machine (ELM) [27], and deep learning (DL) [28] have been widely applied in computational proteomics. Some feature selection techniques such as minimum redundancy maximum relevance feature selection (mRMR) [29], manifold learning (ML) [30], principal component analysis (PCA) [31], and regularized trees [32] have also been developed and were gradually used to obtain optimal features that produce the highest predictive accuracy.

Developing a set of informative parameters to formulate the ion channel samples is also necessary for ion channel prediction. In this paper, only the amino acid, the dipeptide, and tripeptide composition were used to represent ion channels. The physiochemical characteristics [33], overrepresented motifs [34], and functional domains [35] can also be utilized in the field.

Of course, to construct better benchmark dataset which not only contains more sequences but also obeys more objective and strict standards can benefit the study ion channels. Now, with the avalanche of genome and proteome sequences generated in the postgenomic age, many ion channels are available in various sequence, structure, and reference database. Collecting and building these data is the key role in ion channel study.

In the future, we hope that researchers can focus on the three aspects discussed above for developing powerful and efficient predictors of ion channels.

## 6. Summary

This review focused on the development of prediction methods for ion channels in terms of the following issues:

- datasets of ion channel proteins,
- machine learning methods to predict ion channels,
- feature selection techniques to obtain optimal features for ion channel predictions,
- prospect of ion channel predictions by using bioinformatics methods.

## Figures and Tables

**Table 1:** List of databases related with ion channels.

Name	URL
PDB	http://www.rcsb.org/pdb/home/home.do
Uniprot	http://www.uniprot.org
IUPHAR	http://www.iuphar-db.org
LGIC	http://www.ebi.ac.uk/compneur-srv/LGICdb/LGICdb.php
VKCDB	http://vkcdb.biology.ualberta.ca/index.php

**Table 2:** List of three programs.

Name	URL
BLASTClust	http://www.ncbi.nlm.nih.gov/Web/Newsltr/Spring04/blastlab.html
CD-HIT	http://weizhong-lab.ucsd.edu/cd-hit
PISCES	http://dunbrack.fccc.edu/PISCES.php

**Table 3:** Summary of the ion channel prediction tools.

Name	URL
VGIchan	http://www.imtech.res.in/raghava/vgichan/
IonchanPred	http://cobi.uestc.edu.cn/people/hlin/tools/IonchanPred/
VKCPred	http://cobi.uestc.edu.cn/people/hlin/tools/VKCPred/
iVKC-OTC	http://lin.uestc.edu.cn/server/iVKC-OTC/
